# Screening for Mutations in *ABCC8* and *KCNJ11* Genes in Saudi Persistent Hyperinsulinemic Hypoglycemia of Infancy (PHHI) Patients

**DOI:** 10.3390/genes6020206

**Published:** 2015-04-13

**Authors:** Ahmad Adi, Bassam Bin Abbas, Mohamed Al Hamed, Nada Al Tassan, Dana Bakheet

**Affiliations:** 1 College of Medicine, Alfaisal University, P.O. Box 50297, Riyadh 11533, Saudi Arabia; E-Mail: aadi@alumni.alfaisal.edu; 2 Department of Genetics, King Faisal Specialist Hospital and Research Center, P.O Box 3354, Riyadh 11211, Saudi Arabia; E-Mails: hamed@kfshrc.edu.sa (M.A.H.); naltassan@kfshrc.edu.sa (N.A.T.); 3 Department of Pediatrics, King Faisal Specialist Hospital and Research Center, P.O Box 3354, Riyadh 11211, Saudi Arabia; E-Mail: benabbas@kfshrc.edu.sa

**Keywords:** PHHI, persistent hyperinsulinemic hypoglycemia of infancy, polymorphisms, deletion, *ABCC8* gene, *KCNJ11* gene

## Abstract

The autosomal recessive form of persistent hyperinsulinemic hypoglycemia of infancy (PHHI) is associated with mutations in either *ABCC8* or *KCNJ11* genes. In the present study, we describe the clinical features and results of genetic analysis of 13 Saudi Arabian patients with PHHI. Clinically, most patients presented with infantile seizures and/or developmental delay, with a subset of patients who were also found to have abnormal brain imaging and electrophysiological studies. Interestingly no coding pathogenic mutations were identified in these two genes by direct sequencing. However, two splice variants were identified in *ABCC8* gene in two patients, and a large deletion of exons 1-22 of the *ABCC8* gene was identified in three patients. Our data shows that large deletions in *ABCC8* gene are the common genetic mechanism in the Saudi population.

## 1. Introduction

Persistent hyperinsulinemic hypoglycemia of infancy (PHHI) (MIM# 256450), previously known as nesidioblastosis, is a disorder characterized by impaired suppression of pancreatic insulin secretion, resulting in hypoglycemia [1,2,3]. PHHI presents clinically in neonates and infants less than one year of age with seizures and coma. Incidence of PHHI in Saudi Arabia has been estimated to be 1 in 2675, which is much higher than the internationally reported incidence of 1:50,000 [2,4,5,6]. PHHI is diagnosed clinically by having a glucose requirement more than 8mg/kg/min, having a glucagon response increasing glucose by more than 30 mg/dL, absent ketones and insulin:glucose more than 0.3 [1,2,7]. PHHI is generally treated with diazoxide and somatostatin analogue. Therapy tends to fail especially in familial cases [2]. PHHI long term complications include mental retardation, epilepsy, pancreatic insufficiency and obesity [8]. Diabetes mellitus has been proposed to be the definite outcome of PHHI on the long term [2].

From a genetic perspective, while most cases of PHHI are sporadic, genetic variants can either be autosomal dominant or recessive [2]. Autosomal dominant forms display mutations in the enzymes glutamate dehydrogenase (GLUD1) or in glucokinase (GCK) [1,2,9]. Autosomal recessive forms have mutations in one of the subunits of the K-ATP channels in the pancreatic beta cells, most commonly mutations in the sulfonylurea receptor (*ABCC8* or *SUR1*) or the inward rectifying potassium channel (Kir6.2 or *KCNJ11*) [1,2,5,6,9,10,11]. *ABCC8* and Kir6.2 are both located on chromosome 11p15.1 (adjunct genes), with *ABCC8* having 39 exons, which encode 1581 amino acids, and *KCNJ11* having one exon encoding 390 amino acids [10]. Both gene products function in the K-ATP channels that act on glucose-dependent insulin release from pancreatic beta cells. A third type of inheritance is loss of maternal alleles on chromosome 11p15 [1]. Several mutations have been identified and described in both genes, however some reported cases did not have an identified genetic change [5,12,13]. Studies have detected missense and nonsense mutations of both genes in Arabs population [14,15]. Some studies report PHHI associated with Usher syndrome, as genes responsible for normal ear and eye development are located adjacent to the loci of *ABCC8* and *KCNJ11* [10,16].

Utilizing the highly inbred nature of Saudi population and the increased incidence of PHHI, the objective of this study was to identify genetic changes in both *ABCC8* and *KCNJ11* in a cohort of Saudi patients with PHHI. Sequencing of coding exons of both genes was performed, and we present in this paper the genetic changes that were detected in our cohort, as well as clinical data on our patients.

## 2. Experimental Section

### 2.1. Patients and Samples

This study was approved by King Faisal Specialist Hospital and Research Center in Riyadh, Saudi Arabia (KFSHRC) (RAC#2020007). Patients from pediatric endocrinology clinic who were diagnosed with PHHI were enrolled. Three to five milliliters of blood was collected from patients in EDTA tubes for sequencing and genetic analysis.

Medical and clinical features were recorded for each patient, including birth and developmental history. Comorbidities were noted which includes accompanying congenital malformations if any. Laboratory findings and metabolic profile, which include the above mentioned criteria for diagnosis of PHHI, were noted. To exclude other causes as infantile seizures and developmental delay, which could include congenital neurological defects and/or congenital syndromes such as Usher syndrome that may present with deafness, electrophysiological studies (brainstem evoked auditory potential (BEAP), visual evoked potential (VEP), and electroencephalogram (EEG)) were used. Radiological assessment (brain MRI) was used to exclude focal neurological defects. Medical/surgical treatments also included identifying cases.

### 2.2. DNA Extraction

DNA was extracted from peripheral blood samples from 13 PHHI patients using the Gentra Systems PUREGENE DNA Isolation kit according to manufacturer conditions, with approved written informed consent in adherence to institutional and international guidelines.

### 2.3. Sequencing and Screening for Variants in ABCC8 and KCNJ11

Polymerase chain reaction (PCR) was performed using the ABI Prism^®^ Big Dye^®^ Terminator v3.1 Cycle Sequencing Kit as described by the manufacturer. Results were exported in one of several formats for visualization and analysis of sequence was analyzed using SeqMan 6.1 (Lasergene 6 software package, DNASTAR, Inc., Madison, WI, USA). The open reading frame (ORF) of *ABCC8* and *KCNJ11* was sequenced in all patients, and PCR products covered all exons. Primers for both genes are available upon request.

### 2.4. Confirmation of Deletions Using Multiplex Ligation-Dependent Probe Amplification 

*ABCC8* exons were quantified using SALSA MLPA probemix (SALSA MLPA P117 *ABCC8* probemix P117-B, MRC-Holland, Amsterdam, Holland), according to manufacturer conditions. MLPA was performed on patient’s samples and controls. Electrophoresis of amplicons was performed on ABI 3130 genetic analyzer (Applied Biosystems, Grand Island, NY, USA). Data was exported and analyzed using (Coffalyser.Net-01, MRC-Holland, Amsterdam, Netherlands). Relative peak area (RPA) values were compared to references controls. Normalization of peak area of each amplicon was preformed according to manufacture protocol.

## 3. Results

### 3.1. Clinical Assessment

A total of 14 patients were enrolled in this study (12 patients and a pair of twins). There was one family that had two affected individuals, with the rest of the cases being sporadic. All patients presented as neonatal persistent hypoglycemia and were intolerant to fast. Eleven patients out of 14 presented with neonatal seizures and 13 of 14 patients presented with mental retardation and/or developmental delay. All patients were diagnosed with PHHI according to the criteria mentioned in the introduction. Electrophysiological studies (BEAP): four patients had no response suggesting clinical deafness, three were not tested, and seven patients were normal. Electrophysiological studies (VEP): right patients showed normal results, two patients had no response, while four patients did not have the test. EEG showed generalized epileptiform discharges in seven patients, one patient displayed slow background activity, three patients did not have the EEG done, and three patients were normal. Radiological studies using MRI: one patient showed severe brain atrophy, one patient showed delayed myelination, two patients did not have any radiological studies, one patient had a normal CT but the MRI was not done. On the other hand, nine patients had normal brain MRI.

With regards to medical therapy, all patients were placed on diazoxide and/or octreotide. Ten patients were reported to have failed medical therapy, requiring surgical intervention. One of these patients was recommended for surgical intervention; however, it was not done due to family preferences. Four patients were good responders. Table 1 summarizes the clinical features of each patient.

### 3.2. Variants Identified in ABCC8 and KCNJ11

Table 2 and Table 3 summarize the results for coding variants identified in *ABCC8* and *KCNJ11* genes respectively. A total of 20 homozygous variants were identified for *ABCC8*, and four variants for *KCNJ11*. Two homozygous intronic variants in the *ABCC8* (IVS3 +3 G > A and IVS16 −3 C > T) were identified in a couple of patients suspected to affect splicing. Various intronic variants were identified in *ABCC8* follows: IVS3 +77 G > A, IVS4 +14 C > T, IVS6 +87 G > A, IVS11 +51 G > C, IVS11 +53 T > G, IVS11 +73 G > A, IVS18 −51 T > C, IVS18 −65 G > A, IVS23 +17 A > G, IVS33 −27 T > C, IVS34 +62 G > A, IVS38 −40 A > G, IVS38 +54 G > C. Insilco analysis using PolyPhen-2 and mutation taster predicted that most variants in both genes are non-disease causing.

### 3.3. Large Deletions in ABCC8 Gene

MLPA was performed on all patients, 3 patients from different families showed a common deletion that affected exons1-22 of the *ABCC8* gene. These patients are not related. This deletion knocks out the N-terminus of the protein including the first conserved ATP binding domain.

## 4. Discussion

Limitations of our study are related to the availability of the data collected on our patients. Histological data was not done on patients of this study, which represents a possible future approach on PHHI studies in Saudi Arabia. Another limitation of the study that we will need to pay attention to in future studies is better documentation of patient clinical and biochemical profiles. From a clinical point of view, three patients have clinical findings suggestive of deafness, which has been previously reported in the literature, where deletions in the *ABCC8* gene also cause deletions in an adjacent gene *USH1C* associated with hearing loss (Usher syndrome) [10,16]. In terms of biochemical profiles, future studies should include better documentation of acylcarnitines and urine organic acids. This is because *HADHSC* gene mutations have been described in the literature as potential causes of PHHI [17].

Other studies characterized genetic changes that affect another regulatory portion of the potassium channels in beta cells that caused diabetes in affected individuals [18,19]. This is another possibility to investigate in which there may another change that causes hyperinsulinism instead. Genes other than *ABCC8* and *KCNJ11* have been described in the literature, including *UCP2* (in both animal models and human patients) [20].

**Table 1:** Summary of clinical data for the patients in this study.

Patient	Age (at Time of Study Conduction)	Clinical Presentation *	Brainstem Evoked Auditory Potential–BEAP	Visual Evoked Potential–VEP	EEG Findings	Radiological Studies (MRI or CT)	Treatment
1	3 Years	Neonatal seizures, mental retardation, developmental delay	No response bilaterally	Normal	Generalized epileptiform discharges	Normal MRI	Failed medical therapy, surgical intervention performed
2	8 Years	Neonatal seizures	Normal	Normal	Normal	Normal MRI	Responded to medical therapy
3	8 Years	Neonatal seizures, mental retardation, developmental delay	Normal	Normal	Severe abnormal epileptiform discharges	Normal MRI	Failed medical therapy, surgical intervention performed
4	4 Years	Neonatal seizures, mental retardation, developmental delay	Normal	Normal	Slow background activity	Normal MRI	Failed medical therapy, surgical intervention performed
5	11 Years	No additional clinical symptoms	Normal	Normal	Not done	None done	Responded to medical therapy
6	6 Months	Neonatal seizures, developmental delay	Not done	Not done	Generalized epileptiform discharges	Normal MRI	Failed medical therapy, surgical intervention performed
7	14 Years (Twin)	Neonatal seizures, mental retardation, developmental delay	Normal	Normal	Normal	Normal MRI	Failed medical therapy, surgical intervention performed
8	14 Years (Twin)	Neonatal seizures, mental retardation, developmental delay	Normal	Normal	Normal	Normal MRI	Failed medical therapy, surgical intervention performed
9	2 Years	Developmental delay	Not done	Not done	Not done	Normal CT	Responded to medical therapy
10	3 Years	Neonatal seizures, mental retardation, developmental delay	Not done	Not done	Generalized epileptiform discharges	Normal MRI	Responded to medical therapy
11	9 Years	Neonatal seizures, mental retardation, developmental delay, deafness, blindness	No response bilaterally	No response	Generalized epileptiform discharges	MRI: Severe brain atrophy	Failed medical therapy, surgical intervention performed
12	7 Years	Neonatal seizures, mental retardation, developmental delay, deafness, blindness	No response bilaterally	No response	Generalized epileptiform discharges	MRI (brain): Delayed Myelination	Some response to medical therapy - Surgery refused by family
13	6 Years	Neonatal seizures, mental retardation, developmental delay, decreased hearing and vision	No response bilaterally	Normal	Generalized epileptiform discharges	Normal MRI	Failed medical therapy, surgical intervention performed
14	7 Years	Developmental delay	Normal	Not done	Not done	None done	Failed medical therapy, surgical intervention performed

* In addition to neonatal persistent hypoglycemia and Intolerance to fast.

**Table 2:** Coding variants for *ABCC8* gene (the presence of an rs number represents a change that has been reported in the literature previously).

Nucleotide Position	Codon Number	rs Number
c.207 T > C	P69P	rs1048099
c.1812 C > T	H562H	No rs number
c.3819 G > A	R1273R	rs1799859
c.4231 G > T	A1369S	No rs number
c.4717 G > A	V1573V	No rs number

**Table 3:** Coding variants for *KCNJ11* gene (the presence of an rs number represents a change that has been reported in the literature previously).

Nucleotide Position	Codon Number	rs Number
c.67 A > G	K23E	rs5219
c.570 C > T	A190A	rs5218
c.1009 G > A	V337I	rs5215
c.1143 G > A	K381K	rs8175351

Another hypothesis that explains the mutations we report is different genetic composition of different populations. As mentioned before, PHHI is more prevalent in Saudi Arabia than the world population. One of the explanations is the highly inbred nature of the Saudi community, which may explain the emergence of new mutations in *ABCC8*, *KCNJ11*, or other novel genes. This explanation is similar to previous literature, where a study reports novel genetic mutations in *ABCC8* (macrodeletion) that was found in the Spanish population patients, which were not characterized in the literature before [5], three of our patients harbored a common homozygous deletion of exons 1–22. A study also characterized novel mutations in *ABCC8* and *KCNJ11* in the Russian population, which were novel loci for susceptibility to both type II diabetes and altered insulin secretion [21].

Clinically, only 4 patients out of 13 responded to medical treatment, which is consistent with previous report where genetic changes in *ABCC8* and *KCNJ11* did not result in a complete loss of function [22]. There are reports of patients who failed medical therapy, who had *ABCC8* mutations, but not *KCNJ11*, similar to our surgical patients [23,24]. This suggests that *ABCC8* mutations are associated clinically with worse outcome and did not respond to medical therapy than *KCNJ11*. Also, some of our patients had negative test to VEP, which also suggests eye abnormalities as previously reported [10]. These findings suggest that symptoms of PHHI and hearing loss are related both clinically and genetically, and pave the way to better characterization of both diseases. Our patients were not evaluated for *USH1C* genetic changes, which represents a future direction of the study.

Future directions for studying PHHI in Saudi Arabia include utilizing other techniques in scanning for genetic abnormalities; for example, whole genome sequencing, the recruitment of more patients, and also to analyze unaffected individuals in families with PHHI, especially if any is diagnosed with diabetes mellitus. Another future direction would be to recruit adult patients with hyperinsulinemic hypoglycemia that is not due to an insulinoma. This pool of patients has been described before in the literature, but were not found to have mutations in both *ABCC8* and *KCJN11*, which again further reinforces the idea that hyperinsulinemic hypoglycemia can be multifactorial [25,26]. Furthermore, we are only reporting changes in 13 patients. As mentioned earlier, PHHI has a higher incidence in Saudi Arabia than what would be predicted. This represents a future direction of this study in which we would be recruiting more patients and families to increase the power of the study. One consideration for the data above is that the families recruited to the study mostly come from rural backgrounds where consanguinity is prevalent. This rural background could possibly be a source of late-look bias, since PHHI is associated with infant mortality (we are only seeing the cases that survive long enough to be seen at our center). Therefore, a future direction of this study is to have clinical teams recruit and screen patients in rural areas, which will result in a wider cohort and a higher chance of identifying other genetic changes associated with PHHI.

## 5. Conclusions

Mutations that are described above for both *ABCC8* and *KCNJ11* are consistent with previous studies. Studies report a mosaic nonsense mutation in *ABCC8*, as well as nonsense mutations in *KCNJ11* [27,28]. Similar to our study, there was a previous report of splice-site mutations in the *ABCC*8 gene [14]. Nonsense mutations were not identified in our patients, which highlights that PHHI can be caused by a variety of genetic changes. The fact that the mutations above are unlikely to cause disease hints at new possibilities in terms of the pathogenesis of PHHI in Saudi population. A previous study suggested another model of inheritance of uniparental disomy and paternal inheritance of KATP (K-ATP) genes, which produces focal lesions. The same study also proposed a mosaic inheritance pattern for PHHI [27].
